# The Interaction of Sleep and Mood During Recovery from Mild Traumatic Brain Injury

**DOI:** 10.1177/2689288X251377033

**Published:** 2025-09-16

**Authors:** Robin McGee, Melanie A. Montoya, Jason Barber, Keanan J. Joyner, Lindsay D. Nelson, Nancy Temkin, Emerson Wickwire, Geoffrey T. Manley, David M. Schnyer, the TRACK-TBI Investigators

**Affiliations:** ^1^ Department of Psychology, University of Texas at Austin, Austin, Texas, USA.; ^2^ Departments of Neurological Surgery and Biostatistics, University of Washington, Seattle, Washington, USA.; ^3^ Department of Psychology, University of California, Berkeley, Berkeley, California, USA.; ^4^ Departments of Neurosurgery and Neurology, Medical College of Wisconsin, Milwaukee, Wisconsin, USA.; ^5^ Department of Psychiatry, University of Maryland School of Medicine, Baltimore, Maryland, USA.; ^6^ Department of Neurosurgery, University of California, San Francisco, San Francisco, California, USA.

**Keywords:** concussion, depression, insomnia, mild traumatic brain injury, mood

## Abstract

Insomnia and depression are common co-morbidities associated with mild traumatic brain injury (mTBI). Data from Transforming Research and Clinical Knowledge in TBI, a longitudinal cohort study of TBI and orthopedic controls (OTC), were used to examine insomnia trajectories and the temporal relationship between insomnia and depressive symptoms during recovery. mTBI (*n* = 1,557) and OTC (*n* = 226) adult patients with no psychiatric or sleep disorder history were assessed at 2 weeks and 3, 6, and 12 months post-injury, and at three long-term assessments between 2 and 10 years post-injury. Latent class growth analysis identified five insomnia trajectory classes during the first year post-injury, revealing 25% with persistent insomnia, 4% improving, and 71% below the clinical cutoff. A random intercept cross-lagged panel model tested the lagged effects between insomnia and depression. In addition to being longitudinally correlated (φ = 0.74, *p* < 0.001), depressive symptomatology operated as a leading indicator of worsening insomnia from 3 to 6 months post-injury (β = 0.20, *p* = 0.001) across the whole sample. The multigroup model revealed less insomnia (α = −0.31, *p* = 0.006) and depressive symptoms (α = −0.52, *p* < 0.001) in OTC relative to mTBI. From 1 to 5–10 years post-injury, mTBI low insomnia classes remained stable, while the highest class improved moderately (−5.50, 95% confidence interval: −7.84, −3.16, *p* < 0.001). Our findings suggest depressive symptoms may lead to worsening insomnia during the subacute recovery period and that a subset of patients with mTBI may suffer from new-onset insomnia that persists for more than 5 years.

## Introduction

Mild traumatic brain injury (mTBI) represents a major public health burden^1,2^ and has been associated with a host of physical and psychological difficulties, including chronic pain, cognitive impairment, mood, and sleep difficulties.^2–8^ Approximately half of adults report disturbed sleep during recovery from TBI.^2,9,10^ Insomnia, defined as difficulty initiating/maintaining sleep with associated daytime consequences,^11^ is the most common sleep disorder seen in clinical practice. A recent meta-analysis estimated the prevalence of post-mTBI insomnia disorder at 27%, three times the rate of the general population,^12,13^ with a striking 72% of patients experiencing insomnia symptoms at some point during their recovery.^14^ The impact of insomnia in TBI is profound, contributing to functional impairment,^10^ worse post-concussive symptoms,^15^ delayed recovery,^16^ reduced cognitive performance,^17^ and mood disturbances.^8,18–20^ Concerningly, insomnia can persist for a year or more after injury.^21,22^ Various studies have estimated the prevalence of insomnia disorder at 1 year post-injury between 21% and 69.2%.^13,16,23^ Recent data from the Transforming Research and Clinical Knowledge in TBI (TRACK-TBI) study found that 27.8% of participants (*n* = 2,022) reported moderate to severe insomnia symptoms at 12 months post-injury and identified five distinct classes of insomnia symptoms among patients with TBI: from 2 weeks to 12 months, insomnia symptoms resolved for 33.2% of the sample (classes 2 and 4), remained stable for 66% (classes 1 and 3), and worsened for 0.7% (class 5).^13^

Depressive symptoms are also a frequent problem following TBI and have been strongly linked to insomnia.^2,16,24^ Major depressive disorder (MDD) has been reported in up to 53% of patients with TBI^25^ in general and in 14–18% of patients with mTBI, rendering it the most frequent psychiatric diagnosis in patients with mTBI.^26–29^ Moreover, sleep disturbances are one of the nine criteria used to diagnose MDD,^11^ and evidence suggests poor sleep can precede, follow, and amplify depressive symptoms. However, determining the directional influence between these symptoms has proved challenging, and any causal relationships remain unclear.^30,31^

Prior observational studies examining the longitudinal relationship between these variables have been hampered by small sample sizes, mixed TBI severity, long lags between assessments, and inclusion of subjects with prior sleep disorder history. In a small mTBI sample, sleep disturbance in the acute recovery period predicted depressive and anxiety symptoms at 12 months post-injury, but did not examine whether mood symptoms predicted later sleep disturbances.^32^ Theadom et al. reported that poor sleep at baseline was associated with higher levels of anxiety and depression, but not vice versa, from 2 weeks to 12 months.^23^ A mixed-severity TBI study reported that hypersomnia, but not insomnia, at 3 months post-TBI predicted worsened depressive symptoms at 6 months.^33^ A prospective study of predominantly patients with mTBI followed 6 months after injury found that depressive symptoms uniquely predicted future sleep difficulties, yet the authors did not test the opposite direction or control for pre-injury sleep or psychiatric problems.^10^ Previous research implicates insomnia and depression as key outcomes associated with TBI, but more work remains to understand the complex interplay of these symptoms.

Investigations into the temporal relationship of insomnia and depression post-mTBI are confounded by overlapping constructs and high rates of comorbidity^16,34^; however, methodologies employed in non-TBI populations can provide a useful approach.^35–38^ Recently, Zhou and colleagues utilized a random intercept cross-lagged panel model (RI-CLPM), which parses between- and within-subject effects, to assess a large sample (*n* = 1,200) of the general population during the COVID-19 pandemic at 7 timepoints across 1 year. Controlling for baseline levels of depression and insomnia, results revealed a unidirectional relationship with depressive symptoms significantly predicting subsequent insomnia, but not the reverse.^39^ We are not aware of any studies that have employed this methodology in an mTBI population.

Both insomnia and depression represent important treatment targets with the potential to improve TBI outcomes. We aimed to characterize the post-injury trajectory of insomnia symptoms up to 10 years in a large sample of mTBI and orthopedic controls (OTC), excluding individuals with premorbid sleep disorder or psychiatric history, to elucidate the temporal relationship between insomnia and depression to provide insights that could help with improving treatment and management of mTBI.

## Methods

**FIG. 1.**
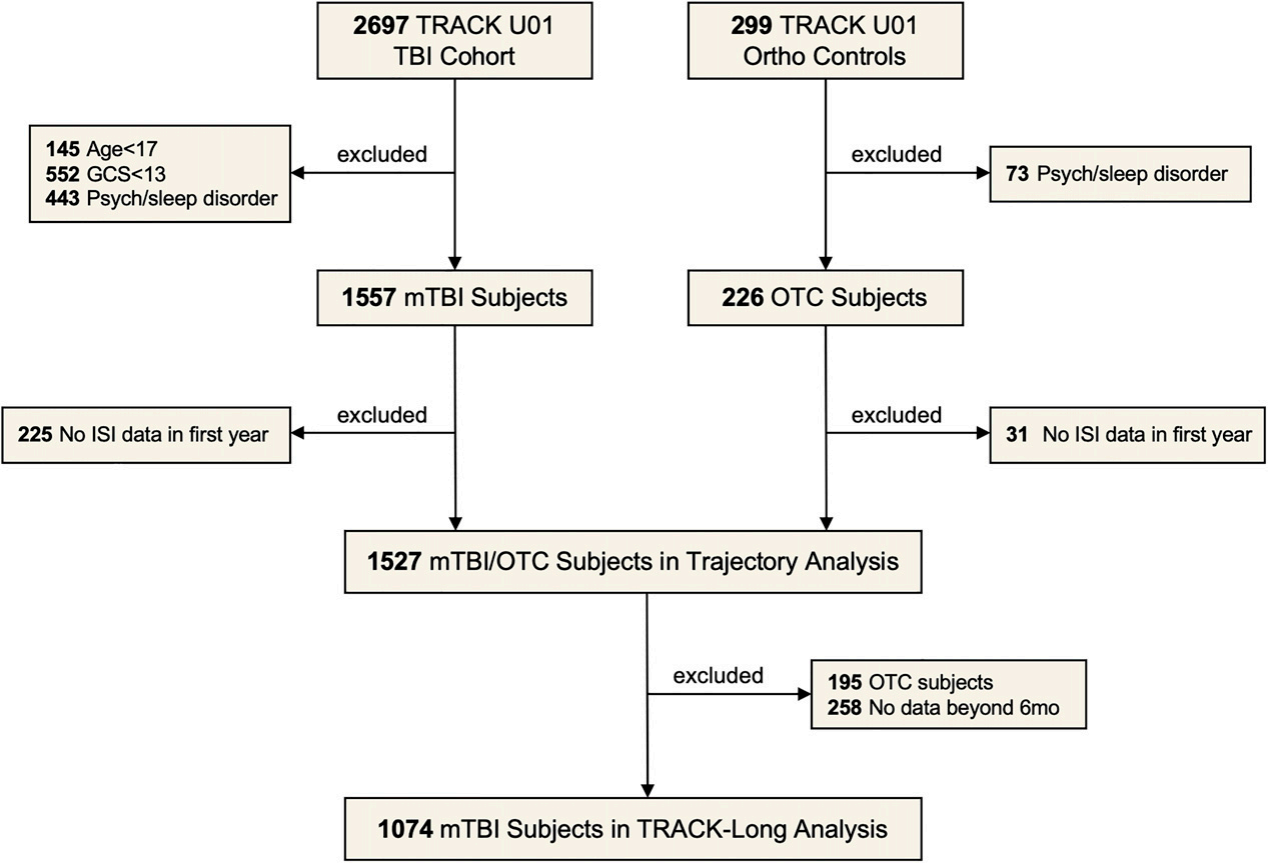
STROBE flow diagram of included subjects. In the post 1 year data, the ISI was not collected for the first 15 months. STROBE, strengthening the reporting of observational studies in epidemiology; ISI, insomnia severity index; mTBI, mild traumatic brain injury; OTC, orthopedic controls; GCS, Glasgow coma scale.

Data for this study were from the prospective, multicenter TRACK-TBI study.^40–42^ Participants were recruited from 18 level 1 trauma centers in the United States within 24 h of a suspected TBI. Inclusion criteria required a clinician-ordered head computed tomography (CT) scan and at least one sign of altered consciousness.^43^ A control group with orthopedic injuries, no signs of TBI, and similar exclusion criteria (described elsewhere)^4^ was also enrolled. Demographic and clinical data were collected at baseline, and outcome measures were collected at 2 weeks and 3, 6, and 12 months following injury, and then at up to three annual phone calls 2–10 years post-injury. We restricted the TBI cohort to adults (≥17 years) with mild TBIs—defined as a Glasgow coma scale (GCS)^44^ score of 13–15 on arrival to the emergency department (ED) regardless of head CT findings—and excluded subjects with any history of psychological or sleep disorders (Fig. 1). This study was reviewed and approved by the institutional review board at the coordinating center (University of California, San Francisco) and at each participating site.

### Outcome measures

The insomnia severity index (ISI) is a 7-item self-report measure assessing insomnia severity and functional impact, with total scores from 0 to 28.^45–47^ A cutoff score of 10 offered optimal sensitivity and specificity for detecting insomnia in a community sample.^47^ It has good psychometric properties^46–48^ and has been used in numerous studies of TBI.^15,49^

The patient health questionnaire-9 (PHQ-9) is a 9-item self-report measure of depressive symptoms, commonly used in clinical settings, with scores from 0 to 27, and a recommended cutoff of 10.^50,51^ It is reliable for identifying depression in adults with TBI.^52–54^

The brief symptom inventory 18 (BSI-18) is an 18-item screen of psychological distress with a Global Severity Index score range of 0–72, and three subscales: somatization, depression, and anxiety. It has been used extensively in TBI populations^55,56^ and has shown excellent reliability and validity in a sample of mixed-severity TBI.^57^

### Statistical analysis

#### Participant characteristics

Differences in characteristics between the mTBI and OTC cohorts were quantified using standardized mean differences, and among the five trajectory groups, they were assessed for statistical significance using Kruskal–Wallis tests for continuous and ordinal variables and Fisher’s exact test for nominal variables. Long-term outcome in the mTBI cohort was modeled using mixed-effects regression fitting random intercepts, with effect sizes estimated parametrically and *p* values non-parametrically using ranks due to the lack of normality in the residuals. All results were interpreted in the context of multiple comparisons using a 5% false discovery rate per the Benjamini–Hochberg method.^58^ Additional analysis was carried out using SPSS Version 26 (IBM Corp., Armonk, NY).

#### Latent class growth analysis

The ISI data were analyzed using latent class growth analysis (LCGA), in which subjects are hypothesized to be clustered into unobserved longitudinal trajectories.^59^ LCGA was selected over latent class mixture modeling (LCMM) used in a prior study^13^ due to its assumption of within-class homogeneity and ability to detect distinct subpopulations. The LCGA models were fit using SAS statistical software version 9.4 (SAS Institute Inc., Cary, NC) that utilized the “proj traj” application.^60^ Multiple candidate trajectory models were estimated, varying both the number (up to eight-class models, as determined by BIC—Bayesian Information Criteria) and shape of the trajectory curves (linear, quadratic, and cubic). Other fit criteria considered included posterior probability, minimum class size, interpretability, and parsimony.^61^ To further winnow the set of candidate models, each class size (5–8) was represented with a single model determined using a backward-elimination approach, in which a full cubic starting model was reduced in steps by removing the highest-order terms within each trajectory one by one until all remaining highest-order terms were statistically significant (*p* < 0.05). All four of these models featured minimum class sizes of 3–7% and minimum posterior probabilities of 68–75%, resulting in the best-fit model being the five-class model (minimum class size of 5% and minimum posterior probability of 75%).

#### Random intercept cross-lagged panel model

RI-CLPM with autoregressive effects was fit using Mplus v.8.1 through the *MplusAutomation* package^62^ in *R* (v. 3.5.1; R Core Team, 2018). RI-CLPMs are particularly desirable beyond a cross-lagged panel model without a random intercept due to the ability of the RI-CLPM to parse between- and within-subject effects, allowing one to draw strong inferences about the temporal sequencing of effects within-subject, while accounting for the overall relations among variables at the between-subject level.^63,64^ Specifically, the model (1) defined random intercepts by fixing all loadings of observed variables across time for that construct (e.g., ISI or PHQ-9 score at 2 weeks, and 3, 6, and 12 months) to 1; (2) defined autoregressive effects by predicting the variable at the current time *t* from the same variable at the previous time point *t* − 1 (e.g., ISI score at 3 months being predicted by ISI score at 2 weeks) for time points 2–4; and (3) defined cross-lagged effects by predicting variable *x* at time *t* from variable *y* at time *t* − 1 (e.g., ISI score at time 2 being predicted by PHQ-9 score at time 1) at the same time as predicting variable *y* at time *t* from variable *x* at time *t* − 1 (e.g., PHQ-9 score at time 2 being predicted by ISI score at time 1) for time points 2–4. Additionally, residual covariances between variables *x* and *y* both at time *t* were specified. Lastly, to examine mean-level differences between mTBI and OTC groups, multigroup structural equation modeling was used, specifying the same RI-CLPM model as above, but freeing the intercepts of the ISI and PHQ-9 variables. Missing data were handled using full information maximum likelihood; parameters were estimated using maximum likelihood with robust standard errors.

## Results

### Participant characteristics

**FIG. 2.**
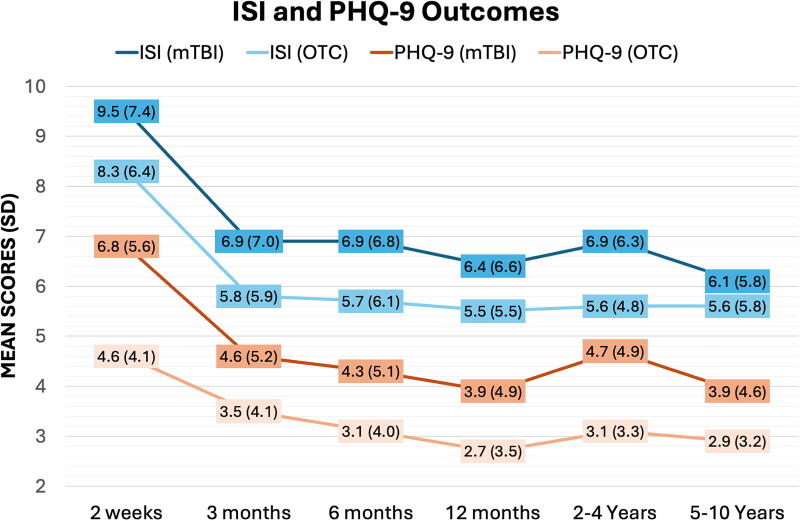
ISI and PHQ-9 mean (SD) scores over time for mTBI and OTC cohorts. ISI, insomnia severity index; PHQ-9, patient health questionnaire-9; SD, standard deviation; mTBI, mild traumatic brain injury; OTC, orthopedic controls.

Demographic, clinical, and injury characteristics for the mTBI and OTC cohorts are presented (Table 1). Means (standard deviation [SD]) of PHQ-9, ISI, and BSI-18 by cohort (Table 2) signal overall decreasing symptoms across time, with mTBI scores higher than OTC at every timepoint (Fig. 2).

**Table 1.** Patient/Injury Characteristics

Injury group	Mild GCS 13–15 (*N* = 1,557)	OTC (*N* = 226)	SMD
			
Age			
Mean (SD)	42.0 (17.9)	40.4 (15.8)	−0.09
Education years			
Mean (SD)	15.2 (11.9)	13.9 (5.8)	−0.12
Unknown	55	5	−0.09
Sex			
Male	1,104 (71%)	161 (71%)	0.01
Female	453 (29%)	65 (29%)	−0.01
Race/ethnicity			
(A) Non-White Hispanic	815 (53%)	103 (47%)	−0.14
(B) Black	280 (18%)	40 (18%)	−0.01
(C) Asian	61 (4%)	8 (4%)	−0.02
(D) White Hispanic	342 (22%)	62 (28%)	0.12
(E) Other	33 (2%)	7 (3%)	0.06
Unknown	26	6	0.06
Insurance			
(A) Insured	856 (59%)	131 (62%)	0.06
(B) Medicare	132 (9%)	16 (8%)	−0.06
(C) Medicaid	152 (10%)	16 (8%)	−0.11
(D) Uninsured	320 (22%)	48 (23%)	0.02
Unknown	97	15	0.02
Employment Status			
(A) Full-time	893 (61%)	151 (69%)	0.20
(B) Part-time	165 (11%)	27 (12%)	0.04
(C) Occasional/special/unemployed	110 (8%)	14 (6%)	−0.04
(D) Retired/disabled/not working	221 (15%)	18 (8%)	−0.23
(E) Student/other	76 (5%)	8 (4%)	−0.07
Unknown	92	8	−0.13
Patient type			
(1) ED discharge	394 (25%)	77 (34%)	0.19
(2) Admit with no ICU	651 (42%)	127 (56%)	0.29
(3) Admit with ICU	512 (33%)	22 (10%)	−0.78
Injury cause			
(A) MVC occupant	489 (31%)	37 (16%)	−0.41
(B) MCC	132 (8%)	27 (12%)	0.11
(C) MVC (cyclist or pedestrian)	245 (16%)	17 (8%)	−0.31
(D) Fall	408 (26%)	70 (31%)	0.10
(E) Assault	101 (6%)	3 (1%)	−0.45
(F) Other/unknown	182 (12%)	72 (32%)	0.43
Initial CT			
Negative	934 (62%)	—	
Positive/suspected	568 (38%)	—	
Unknown	55	—	
TBI history			
None	1,151 (81%)	183 (88%)	0.18
Yes without hospitalization	165 (12%)	19 (9%)	−0.08
Yes with hospitalization	106 (7%)	7 (3%)	−0.21
Unknown	135	17	−0.04
Loss of consciousness			
No	208 (14%)	—	
Yes/suspected	1,271 (86%)	—	
Unknown	78	—	

GCS, Glasgow coma scale; OTC, orthopedic controls; SMD, standardized mean difference; SD, standard deviation; ICU, intensive care unit; MVC, motor vehicle collision; MCC, motorcycle collision; TBI, traumatic brain injury.

**Table 2.** Means (SD) Scores on PHQ-9, ISI, and BSI-18 for mTBI and OTC Cohorts Over 10 Years

	2 weeks	3 months	6 months	12 months	2–4 years	5–10 years
ISI						
Count						
Mild	1,217	1,136	1,065	990	139	376
OTC	177	164	150	142	62	39
Mean (SD)						
Mild	9.5 (7.4)	6.9 (7.0)	6.9 (6.8)	6.4 (6.6)	6.9 (6.3)	6.1 (5.8)
OTC	8.3 (6.4)	5.8 (5.9)	5.7 (6.1)	5.5 (5.5)	5.6 (4.8)	5.6 (5.8)
* N* (%) ≥10						
Mild	551 (45%)	356 (31%)	314 (29%)	260 (26%)	46 (33%)	90 (24%)
OTC	74 (42%)	41 (25%)	35 (23%)	29 (20%)	13 (21%)	8 (21%)
PHQ-9						
Count						
Mild	1,216	1,135	1,063	992	449	476
OTC	178	164	149	142	117	39
Mean (SD)						
Mild	6.8 (5.6)	4.6 (5.2)	4.3 (5.1)	3.9 (4.9)	4.7 (4.9)	3.9 (4.6)
OTC	4.6 (4.1)	3.5 (4.1)	3.1 (4.0)	2.7 (3.5)	3.1 (3.3)	2.9 (3.2)
* N* (%) ≥10						
Mild	332 (27%)	173 (15%)	158 (15%)	132 (13%)	72 (16%)	55 (12%)
OTC	24 (13%)	15 (9%)	9 (6%)	7 (5%)	5 (4%)	2 (5%)
BSI-18 global						
Count						
Mild	1,223	1,141	1,065	991	448	475
OTC	179	164	150	142	117	39
Mean (SD)						
Mild	11.7 (11.8)	8.0 (10.6)	7.8 (10.8)	7.1 (10.1)	2.5 (3.2)	2.0 (2.9)
OTC	7.0 (7.2)	5.5 (7.6)	5.2 (7.7)	4.9 (6.8)	1.7 (2.4)	1.7 (2.8)
BSI-18 depression						
Count						
Mild	1,223	1,141	1,065	991	448	475
OTC	179	164	150	142	117	39
Mean (SD)						
Mild	3.2 (4.3)	2.6 (4.2)	2.6 (4.2)	2.4 (4.0)	2.8 (3.7)	2.2 (3.3)
OTC	2.2 (3.1)	2.0 (3.3)	1.7 (3.2)	1.5 (2.5)	1.9 (2.9)	1.2 (2.3)
BSI-18 anxiety						
Count						
Mild	1,223	1,141	1,065	991	448	475
OTC	179	164	150	142	117	39
Mean (SD)						
Mild	3.6 (4.6)	2.6 (4.1)	2.6 (4.2)	2.3 (3.8)	8.3 (9.6)	6.4 (8.4)
OTC	1.7 (2.8)	1.6 (2.7)	1.6 (3.0)	1.5 (2.7)	5.5 (6.6)	4.0 (5.3)
BSI-18 somatic						
Count						
Mild	1,223	1,141	1,065	991	448	475
OTC	179	164	150	142	117	39
Mean (SD)						
Mild	4.9 (4.6)	2.8 (3.6)	2.6 (3.6)	2.4 (3.5)	3.0 (4.0)	2.2 (3.2)
OTC	3.0 (3.1)	2.0 (2.7)	1.9 (2.8)	2.0 (2.8)	1.9 (2.6)	1.1 (2.1)

SD, standard deviation; PHQ-9, patient health questionnaire-9; ISI, insomnia severity index; BSI-18, brief symptom inventory 18; mTBI, mild traumatic brain injury; OTC, orthopedic controls.

### LCGA results

**FIG. 3.**
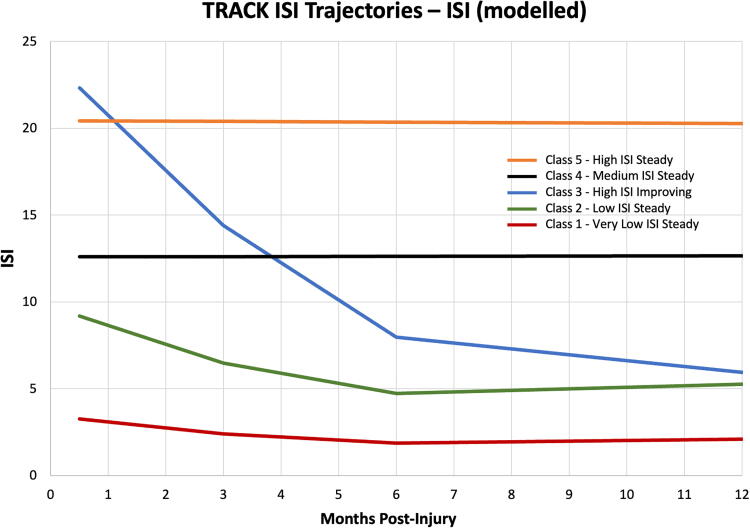
Five-class model of trajectories of insomnia 1 year post-injury (mTBI and OTC). Class 1 (545 of 1,527 [35.7%]) and class 2 (543 of 1,527 [35.6%]) reported low insomnia symptoms below the clinical cutoff of 10. Class 3 (60 of 1,527 [3.9%]) initially reported high levels of insomnia that resolved over the 12-month period. Class 4 (255 of 1,527 [16.7%]) reported medium persistent insomnia, and class 5 (124 of 1,527 [8.1%]) reported high persistent insomnia throughout the 12 months. mTBI, mild traumatic brain injury; OTC, orthopedic controls.

The data for the trajectory analysis were best fit by an LCGA five-class model based on fit parameters (BIC, −14651) and sufficient posterior probabilities in all classes (>75%). The five-class model identified 25% with persistent insomnia, 4% improving, and 71% below the ISI clinical cutoff of 10 (Fig. 3). Demographic and injury characteristics and distribution of mTBI and OTC across classes are reported (Table 3).

**Table 3.** Patient/Injury Characteristics by ISI Trajectory Class

	ISI 12-month trajectory (model #5) Row %s					
	Very low ISI steady *N* = 545 (35.7%)	Low ISI steady *N* = 543 (35.6%)	High ISI improving *N* = 60 (3.9%)	Medium ISI steady *N* = 255 (16.7%)	High ISI steady *N* = 124 (8.1%)	Sig.
Injury group						
mTBI	467 (35%)	466 (35%)	55 (4%)	226 (17%)	118 (9%)	0.018
OTC	78 (40%)	77 (39%)	5 (3%)	29 (15%)	6 (3%)	
Age						
Mean (SD)	42.0 (19.2)	40.8 (17.2)	39.8 (15.3)	41.5 (15.6)	38.6 (13.5)	0.708
Education years						
Mean (SD)	14.4 (8.5)	14.8 (8.9)	14.5 (10.1)	14.0 (8.5)	12.9 (2.5)	<0.001
Sex						
Male	395 (37%)	382 (36%)	42 (4%)	168 (16%)	86 (8%)	0.451
Female	150 (33%)	161 (35%)	18 (4%)	87 (19%)	38 (8%)	
Race/ethnicity						
(A) White Hispanic	304 (38%)	307 (39%)	16 (2%)	114 (14%)	49 (6%)	<0.001
(B) Black	68 (25%)	79 (29%)	22 (8%)	65 (24%)	42 (15%)	
(C) Asian	25 (41%)	29 (48%)	1 (2%)	5 (8%)	1 (2%)	
(D) Non-White Hispanic	134 (38%)	111 (32%)	19 (5%)	58 (16%)	30 (9%)	
(E) Other	10 (29%)	11 (31%)	2 (6%)	10 (29%)	2 (6%)	
Unknown	4 (31%)	6 (46%)	0 (0%)	3 (23%)	0 (0%)	
Insurance						
(A) Insured	315 (35%)	328 (36%)	28 (3%)	163 (18%)	66 (7%)	<0.001
(B) Medicare	61 (51%)	40 (33%)	5 (4%)	10 (8%)	4 (3%)	
(C) Medicaid	40 (28%)	42 (29%)	6 (4%)	34 (24%)	21 (15%)	
(D) Uninsured	114 (36%)	115 (36%)	17 (5%)	43 (14%)	29 (9%)	
Unknown	15 (33%)	18 (39%)	4 (9%)	5 (11%)	4 (9%)	
Employment status						
(A) Full-time	317 (34%)	339 (37%)	40 (4%)	157 (17%)	73 (8%)	0.009
(B) Part-time	61 (33%)	74 (40%)	2 (1%)	29 (16%)	17 (9%)	
(C) Occasional/special/unemployed	35 (34%)	25 (24%)	5 (5%)	19 (18%)	20 (19%)	
(D) Retired/disabled/not working	85 (42%)	67 (33%)	8 (4%)	34 (17%)	10 (5%)	
(E) Student/other	32 (42%)	28 (36%)	2 (3%)	12 (16%)	3 (4%)	
Unknown	15 (45%)	10 (30%)	3 (9%)	4 (12%)	1 (3%)	
Patient type						
(1) ED discharge	153 (36%)	150 (36%)	20 (5%)	63 (15%)	34 (8%)	0.849
(2) Admit with no ICU	234 (35%)	245 (36%)	25 (4%)	120 (18%)	54 (8%)	
(3) Admit with ICU	158 (37%)	148 (34%)	15 (3%)	72 (17%)	36 (8%)	
Injury cause						
(A) MVC occupant	132 (30%)	150 (34%)	33 (7%)	80 (18%)	48 (11%)	<0.001
(B) MCC	45 (34%)	49 (37%)	6 (5%)	23 (17%)	10 (8%)	
(C) MVC (cyclist or pedestrian)	83 (36%)	98 (43%)	5 (2%)	33 (14%)	10 (4%)	
(D) Fall	168 (41%)	139 (34%)	8 (2%)	74 (18%)	23 (6%)	
(E) Assault	26 (29%)	29 (32%)	2 (2%)	17 (19%)	16 (18%)	
(F) Other/unknown	91 (41%)	78 (35%)	6 (3%)	28 (13%)	17 (8%)	
Initial CT						
Negative	271 (32%)	293 (35%)	48 (6%)	147 (17%)	88 (10%)	<0.001
Positive/suspected	189 (40%)	173 (36%)	8 (2%)	79 (17%)	27 (6%)	
Unknown	85 (42%)	77 (38%)	4 (2%)	29 (14%)	9 (4%)	
TBI history						
None	443 (38%)	431 (37%)	37 (3%)	183 (16%)	85 (7%)	<0.001
Yes without hospitalization	51 (31%)	53 (32%)	8 (5%)	31 (19%)	21 (13%)	
Yes with hospitalization	23 (23%)	31 (30%)	9 (9%)	24 (24%)	15 (15%)	
Unknown	28 (34%)	28 (34%)	6 (7%)	17 (21%)	3 (4%)	
Loss of consciousness						
No	145 (39%)	140 (38%)	11 (3%)	54 (15%)	20 (5%)	0.056
Yes/suspected	386 (35%)	382 (35%)	49 (4%)	180 (16%)	102 (9%)	
Unknown	14 (24%)	21 (36%)	0 (0%)	21 (36%)	2 (3%)	

ISI, insomnia severity index; mTBI, mild traumatic brain injury; OTC, orthopedic controls; SD, standard deviation; ICU, intensive care unit; MVC, motor vehicle collision; MCC, motorcycle collision.

### RI-CLPM results

**FIG. 4.**
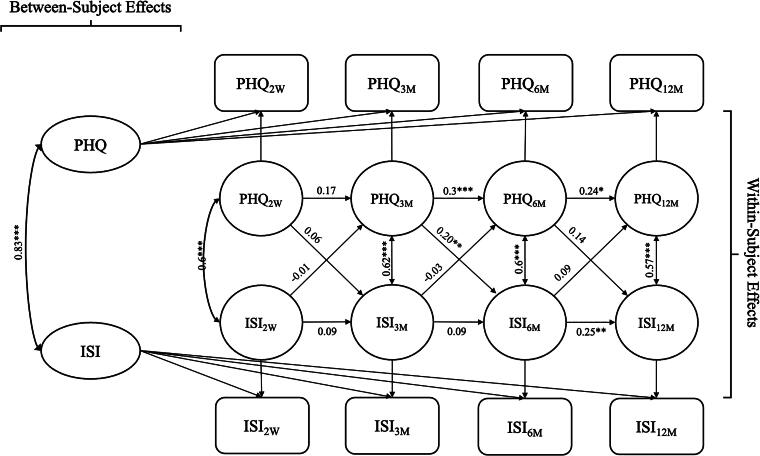
Random intercept cross‐lagged panel model of insomnia and depressive symptoms 1 year post-injury (mTBI and OTC). Global fit parameters: *Χ*^2^(9) = 28.49, *p* < 0.001; CFI = 0.99, TLI = 0.99; RMSEA = 0.04. Variables in rectangles are observed variables, and variables in circles are latent variables. Standardized betas are presented. *p < 0.05, **p < 0.01, ***p < 0.001. Of note, the residual covariance between variables at time point 1 is not grouped with the other time points 2–4 because variables at time point 1 are “full variance” representations, whereas time points 2–4 represent residual variance once accounting for autoregressive and cross-lagged effects. mTBI, mild traumatic brain injury; OTC, orthopedic controls; CFI, comparative fit index; TLI, Tucker–Lewis index; RMSEA, root mean square error of approximation; 2W, 2 weeks; 3M, 3 months; 6M, 6 months; 12M, 12 months.

The full-sample RI-CLPM of ISI and PHQ-9 evidenced excellent global fit (Fig. 4). The random intercepts of the ISI and PHQ-9 were significantly positively associated (*β* = 0.83, *p* < 0.001), and the residual covariances within timepoint were highly correlated (*β*s = 0.57–0.62, all *p*s < 0.001). These results demonstrate a strong relationship between the ISI and PHQ-9 across time. Next, the cross-lagged parameters of the ISI and PHQ-9 predicting each other across time were examined and revealed a significant cross-lagged prediction of the ISI at 6 months by the PHQ-9 at 3 months (*β* = 0.20, *p* = 0.008). The cross-lagged effects of PHQ-9 on ISI from 2 weeks to 3 months (*β* = 0.06, *p* = 0.35) and 6 months to 12 months (*β* = 0.14, *p* = 0.08) were not significant. When the BSI-18 was substituted for the PHQ-9, the same directional pattern emerged from 3 to 6 months (*β* = 0.16, *p* = 0.03). Next, a multigroup model was specified, freeing intercepts for the ISI and PHQ-9 across mTBI and OTC groups, which illustrated that, relative to the mTBI group, the mean of the random intercepts for the OTC group was lower for both the ISI (α = −0.31, *p* = 0.006) and PHQ-9 (α = −0.52, *p* < 0.001).

### Long-term results

ISI, PHQ-9, and BSI-18 means (SDs) by trajectory classes at 2–4 and 5–10 years post-injury are presented (Table 4). Due to small sample sizes in the 2–10 year data (class 5: 2–4 year OTC *n* = 0; 5–10 year OTC *n* = 1), OTC were excluded from the long-term comparisons. Classes 1–3 showed no clinically significant change, while ISI and PHQ-9 scores in classes 4–5 declined significantly from 1 to 2–4 years and 1 to 5–10 years post-injury, though ISI scores remained near or above the clinical cutoff.

**Table 4.** TRACK LONG Assessments by ISI Trajectory (mTBI Cohort)

	ISI 12-month trajectory				
	(1) Very low ISI steady (*N* = 375)	(2) Low ISI steady (*N* = 386)	(3) High ISI improving (*N* = 44)	(4) Medium ISI steady (*N* = 184)	(5) High ISI steady (*N* = 85)
ISI^a^	*p* < 0.001^*^	*p* = 0.236	*p* = 0.246	*p* < 0.001^*^	*p* < 0.001^*^
1 year Mean (SD), 95% CI, *N*	1.5 (2.0) [1.3, 1.7] *N* = 344	5.1 (3.8) [4.7, 5.5] *N* = 355	6.0 (4.4) [4.6, 7.3] *N* = 42	12.7 (4.8) [12.0, 13.4] *N* = 172	20.3 (4.4) [19.4, 21.3] *N* = 77
2–4 years Mean (SD), 95% CI, *N*	3.9 (4.5) [2.6, 5.2] *N* = 47	6.4 (4.9) [5.1, 7.7] *N* = 52	8.4 (9.0) [1.2, 15.6] *N* = 6	10.6 (7.4) [7.7, 13.5] *N* = 25	13.8 (6.7) [8.8, 18.7] *N* = 7
5–10 years Mean (SD), 95% CI, *N*	3.1 (3.6) [2.5, 3.8] *N* = 135	6.0 (4.7) [5.2, 6.8] *N* = 131	9.4 (7.1) [5.9, 12.9] *N* = 16	8.8 (6.4) [7.3, 10.3] *N* = 70	14.8 (5.9) [12.4, 17.3] *N* = 22
1 to 2–4 year change 95% CI, *p* value	2.38 (1.53, 3.24) *p* < 0.001^*^	1.24 (0.04, 2.44) *p* = 0.173	2.46 (−2.46, 7.38) *p* = 0.720	−2.10 (−4.44, 0.25) *p* = 0.003^*^	−6.55 (−10.37, −2.73) *p* = 0.003^*^
1 to 5–10 year change 95% CI, *p* value	1.63 (1.07, 2.19) *p* < 0.001^*^	0.84 (0.01, 1.67) *p* = 0.211	3.45 (0.14, 6.77) *p* = 0.095	−3.92 (−5.48, −2.37) *p* < 0.001^*^	−5.50 (−7.84, −3.16) *p* < 0.001^*^
2–4 to 5–10 year change 95% CI, *p* value	−0.75 (−1.68, 0.18) *p* = 0.270	−0.40 (−1.73, 0.93) *p* = 0.650	0.99 (−4.41, 6.39) *p* = 0.480	−1.83 (−4.38, 0.72) *p* = 0.228	1.06 (−3.14, 5.25) *p* = 0.483
PHQ-9	*p* < 0.001^*^	*p* = 0.102	*p* = 0.059	*p* = 0.007^*^	*p* < 0.001^*^
1 year Mean (SD), 95% CI, *N*	1.1 (1.8) [0.9, 1.2] *N* = 345	3.2 (3.4) [2.8, 3.5] *N* = 356	4.3 (4.7) [2.9, 5.8] *N* = 42	7.3 (5.1) [6.6, 8.1] *N* = 172	12.3 (5.7) [11.0, 13.6] *N* = 77
2–4 years Mean (SD), 95% CI, *N*	2.8 (3.5) [2.2, 3.4] *N* = 144	4.2 (4.5) [3.5, 4.9] *N* = 158	3.7 (3.8) [1.9, 5.5] *N* = 17	7.4 (5.4) [6.2, 8.5] *N* = 87	8.9 (5.6) [7.0, 10.8] *N* = 32
5–10 years Mean (SD), 95% CI, *N*	1.7 (2.4) [1.4, 2.1] *N* = 178	3.9 (3.9) [3.3, 4.4] *N* = 164	7.6 (6.4) [4.9, 10.3] *N* = 21	6.2 (5.7) [4.9, 7.4] *N* = 80	8.3 (5.4) [6.4, 10.2] *N* = 31
1 to 2–4 year change 95% CI, *p* value	1.73 (1.26, 2.21) *p* < 0.001^*^	1.00 (0.29, 1.72) *p* = 0.066	−0.60 (−3.49, 2.29) *p* = 0.721	0.06 (−1.32, 1.43) *p* = 0.357	−3.38 (−5.72, −1.05) *p* = 0.003^*^
1 to 5–10 year change95% CI, *p* value	0.68 (0.24, 1.12) *p* = 0.004^*^	0.70 (−0.01, 1.40) *p* = 0.108	3.26 (0.57, 5.95) *p* = 0.033	−1.15 (−2.56, 0.27) *p* = 0.002^*^	−3.98 (−6.34, −1.62) *p* = 0.001^*^
2–4 to 5–10 year change, 95% CI, *p* value	−1.05 (−1.59, −0.52) *p* = 0.001^*^	−0.30 (−1.14, 0.53) *p* = 0.830	3.86 (0.58, 7.14) *p* = 0.040	−1.20 (−2.82, 0.41) *p* = 0.048	−0.59 (−3.40, 2.21) *p* = 0.662
BSI-18 global (raw)	*p* < 0.001^*^	*p* = 0.041	*p* = 0.290	*p* = 0.002^*^	*p* = 0.007^*^
1 year Mean (SD), 95% CI, *N*	2.3 (3.9) [1.9, 2.7] *N* = 343	5.5 (7.2) [4.8, 6.3] *N* = 356	6.7 (9.3) [3.9, 9.5] *N* = 42	13.0 (11.5) [11.3, 14.7] *N* = 172	23.2 (14.3) [20.0, 26.3] *N* = 78
2–4 years Mean (SD), 95% CI, *N*	5.0 (6.3) [3.9, 6.0] *N* = 143	7.0 (7.8) [5.7, 8.2] *N* = 158	7.6 (8.6) [3.5, 11.6] *N* = 17	13.4 (12.0) [10.9, 15.9] *N* = 87	16.8 (13.1) [12.2, 21.3] *N* = 32
5–10 years Mean (SD), 95% CI, *N*	3.0 (4.3) [2.4, 3.7] *N* = 178	6.1 (6.5) [5.1, 7.1] *N* = 163	11.3 (12.0) [6.2, 16.4] *N* = 21	9.3 (10.6) [7.0, 11.7] *N* = 80	16.6 (12.9) [12.0, 21.1] *N* = 31
1 to 2–4 year change 95% CI, *p* value	2.67 (1.77, 3.57) *p* < 0.001^*^	1.44 (0.09, 2.79) *p* = 0.016^*^	0.88 (−4.82, 6.58) *p* = 0.471	0.37 (−2.59, 3.32) *p* = 0.555	−6.37 (−12.07, −0.67) *p* = 0.012^*^
1 to 5–10 year change, 95% CI, *p* value	0.73 (−0.11, 1.56) *p* = 0.071	0.57 (−0.76, 1.90) *p* = 0.138	4.60 (−0.69, 9.90) *p* = 0.121	−3.68 (−6.72, −0.64) *p* < 0.001^*^	−6.60 (−12.36, −0.84) *p* = 0.010^*^
2–4 to 5–10 year change 95% CI, *p* value	−1.95 (−2.96, −0.93) *p* = 0.001^*^	−0.87 (−2.45, 0.70) *p* = 0.421	3.73 (−2.74, 10.19) *p* = 0.521	−4.04 (−7.52, −0.56) *p* = 0.009^*^	−0.23 (−7.07, 6.61) *p* = 0.947

^a^ The ISI was not part of the TRACK Long assessment for the first 15 months of data collection.

^*^ *p*-Value remains statistically significant (*p* < 0.05) when evaluating in the context of multiple comparisons.

ISI, insomnia severity index; mTBI, mild traumatic brain injury; SD, standard deviation; CI, confidence interval; PHQ-9, patient health questionnaire-9; BSI-18, brief symptom inventory 18.

## Discussion

In this longitudinal study of mTBI and OTC up to 10 years post-injury, we identified five recovery trajectories in the first year post-injury that largely align with previous findings.^13^ Our results indicated that these classes held even with the inclusion of trauma controls and the exclusion of participants with pre-injury psychiatric and sleep disorders. Over the 2–10 year follow-up, the low sleep disturbance classes were relatively stable, while those with higher symptom distress at 1 year demonstrated steady improvement across the long-term follow-up period. Modeling the relationship of sleep and mood symptoms 1 year post-injury, we found that depressive symptoms predicted insomnia from 3 to 6 months post-injury, but not the reverse.

Our findings substantiate and expand upon previous work demonstrating persistent insomnia in a subset of patients with mTBI. At 12 months, classes 4 and 5 (26% of the mTBI sample) exceeded the ISI threshold for significant sleep disturbance, which aligns with other reported prevalence rates.^23^ However, by integrating trauma controls and excluding prior psychiatric and sleep disorder history, we were better able to control for injury and premorbid factors than previous studies. The higher distribution of mTBI in the medium and high steady classes 4 and 5 (26% mTBI vs. 18% OTC) and lower distribution in the low ISI classes 1 and 2 (79% OTC vs. 70% mTBI) suggest that TBI factors may be related to the incidence of clinically significant levels of insomnia post-injury. In contrast to a prior analysis,^13^ our model did not identify a worsening class. This discrepancy could be linked to that study’s inclusion of participants with pre-existing sleep disorders and psychiatric illnesses, who comprised 20% and 33%, respectively, of the worsening classes in that study, or mixed TBI severity in that sample. The difference in class membership for the low symptom groups (60% in the prior study^13^ vs. 36% in this study) likely reflects distinctions in modeling. LCMM, used in the prior study, allows within-class heterogeneity and may better capture clinical variability but can obscure discrete subpopulation structures that LCGA is designed to detect.

Our long-term analyses 1–10 years post-injury demonstrate that, on average, those in classes 1–3 remained below the clinical cutoff for insomnia, and classes 4 and 5 showed modest improvement. This is reassuring for patients with mTBI suffering with insomnia, as class 4 ISI scores (17% of our sample) dropped below the ISI clinical cutoff after 5–10 years. However, at that same timepoint, class 5 (8% of our sample) remained above the clinical cutoff (*M* = 14.8), suggesting that a subgroup of patients with mTBI may experience a chronic level of insomnia that persists for up to a decade.

The cross-lagged analysis offers a novel temporal inference on the interaction of insomnia and depressive symptoms in the subacute phase of recovery. Depressive symptoms at 3 months emerged as a significant predictor of worsening insomnia at 6 months, and a similar, though weaker, association was observed from 6 to 12 months. The effect size from 3 to 6 months was indicative of a strong cross-lagged relationship,^65^ suggesting the first 3 months post-injury may be a critical time to prevent and minimize depression. Both mTBI and OTC cohorts independently displayed this same longitudinal interplay, although the random intercepts model group-differences results confirmed that patients with mTBI face a greater burden of insomnia and mood symptoms than OTC in the first year of recovery.

Notably, we identified no significant cross-lagged effect of insomnia on depression. This unidirectional relationship of depression leading to insomnia was somewhat surprising given the evidence supporting the reverse and bidirectionality,^23,31,32^ but several factors may account for this. First, the lagged effect of sleep deprivation on mood may operate on a smaller time scale than the timepoints in this study. Indeed, sleep disturbances’ impact on depressive symptoms has been shown to occur in as little as 2–4 days,^66^ effects which would be incorporated into the contemporaneous correlations in our model. Conversely, the time frame could be longer, as earlier studies assessing the risk of poor sleep on psychiatric disturbances utilized a 6- to 12-month lag.^23,32^ Finally, there is substantial sample heterogeneity in existing TBI studies, and our findings are consistent with a recent large-scale study in the general population.^39^

Current evidence suggests that our findings may be generalizable beyond the CT-ordered population. Post-TBI mood outcomes are not robustly associated with TBI severity or CT status, as adults with GCS scores of 13–15 have comparable mood outcomes following TBI, regardless of whether a head CT was performed^67^ or the CT scan was positive or negative.^68^ Moreover, symptom burden was either greater in milder cases^69^ or not significantly associated with severity.^70^ However, future work should explore the possible moderating effect of TBI severity on the pattern of insomnia and depressive symptom interaction post-recovery.

The key strengths of this study include a large, well-characterized sample that included OTC and excluded individuals with prior psychiatric and sleep disorder history, a prospectively collected dataset spanning 10 years, and the use of a robust modeling enabling us to identify patterns of recovery as well as draw strong temporal inferences from our time-nested data. The RI-CLPM’s high random intercept confirmed the suitability of this model over a traditional cross-lagged model due to its capability to distinguish within-subject and between-subject effects.

While the findings are important, there are notable limitations with this study. First, self-report questionnaires are vulnerable to bias and may be compounded by injury-related factors.^71,72^ Future work should explore the use of objective sleep assessment methods, such as polysomnography, to substantiate our findings. Additionally, the use of only four data collection timepoints within 1 year may have hindered our ability to detect interrelationships occurring within smaller temporal windows. Our models also did not incorporate other domains such as pain, which may interplay with mood and insomnia symptoms to produce unique classes of maladaptive phenotypes. Further, this dataset did not include details on patient treatments, interventions, therapeutics, or agents that could have worsened insomnia symptoms. Finally, our long-term findings may be impacted by regression to the mean, participant attrition, and reduced sample sizes in the 2–10 year phone calls.

Although many patients with mTBI who develop post-injury sleep disturbances continue to recover years later, our study demonstrated that insomnia remains a persistent problem for a subset of patients with mTBI, is associated with (and may be driven by) adverse neuropsychiatric outcomes, and is experienced more profoundly by those with brain injuries compared with other injuries. This underscores the importance of early identification and treatment of depressive symptoms to prevent escalating sleep difficulties and highlights the urgent need to develop targeted therapies to improve sleep and mood outcomes for patients with TBI.

## Abbreviations Used

BIC: Bayesian Information Criteria
BSI-18: brief symptom inventory 18
CFI: comparative fit index
CI: confidence interval
CT: computed tomography
ED: emergency department
GCS: Glasgow coma scale
ICU: intensive care unit
ISI: insomnia severity index
LCGA: latent class growth analysis
LCMM: latent class mixture modeling
MCC: motorcycle collision
MDD: major depressive disorder
MTBI: mild traumatic brain injury
MVC: motor vehicle collision
OTC: orthopedic trauma control
PHQ-9: patient health questionnaire-9
RI CLPM: random intercept cross-lagged panel model
RMSEA: root mean square error of approximation
SD: standard deviation
SMD: standardized mean difference
STROBE: strengthening the reporting of observational studies in epidemiology
TBI: traumatic brain injury
TLI: Tucker-Lewis index
TRACK TBI: Transforming Research and Clinical Knowledge in Traumatic Brain Injury
